# The efficacy and cerebral hemodynamics mechanisms of acupuncture on the posterior circulation ischemic stroke with vertigo: study protocol for a multicenter, randomized, controlled trial

**DOI:** 10.3389/fneur.2026.1768559

**Published:** 2026-04-21

**Authors:** Meng Gong, Pei Li, Renyan Xiao, Lina Pang, Xiangyin Ye, Shufang Li, Taijun Jiang, Hong Guo, Hongling Duan, Xuemei Deng, Song Jin

**Affiliations:** 1 School of Acupuncture and Tuina, Chengdu University of Traditional Chinese Medicine, Chengdu, China; 2 School of Health Preservation and Rehabilitation, Chengdu University of Traditional Chinese Medicine, Chengdu, China; 3 Department of Rehabilitation Medicine, Traditional Chinese Medicine Hospital of Longquanyi, Chengdu, China; 4 Department of Rehabilitation, Hospital of Chengdu University of Traditional Chinese Medicine, Chengdu, China; 5 Physician in Charge, Chengdu Shuangliu Hospital of Traditional Chinese Medicine, Chengdu, China

**Keywords:** acupuncture, PCIS, randomized controlled trial, study protocol, vertigo

## Abstract

**Introduction:**

Posterior circulation ischemic stroke (PCIS) with vertigo is a common central vertigo disease that significantly hinders patients’ motivation for rehabilitation and increases the recurrence rate and mortality among stroke patients due to recurrent episodes of vertigo. Acupuncture has shown promising therapeutic effects in the treatment of PCIS with vertigo, but its underlying mechanisms remain unclear. This study is designed to investigate the impact of acupuncture on cerebral hemodynamics and brain structure in PCIS patients vertigo, and to evaluate its clinical effectiveness in managing this condition.

**Methods and design:**

This is a multicenter, randomized, controlled trial that will randomly allocate 234 participants in a 1:1:1 ratio to manual acupuncture group, sham acupuncture group, or western medication group. This trial is primarily designed as an explanatory trial, with the primary comparison being manual acupuncture vs. sham acupuncture to evaluate the specific efficacy of acupuncture; comparisons between acupuncture and western medication are regarded as secondary comparative effectiveness analyses. All groups will receive standard secondary stroke prevention. Manual and sham acupuncture will be administered five times weekly for 3 weeks (30 min/session), while the western medication group will receive oral betahistine mesilate tablets three times daily, 5 days per week, for 3 weeks. Assessments will be conducted at baseline (Week 0), post-treatment (Week 3), and at Week 11. The primary outcome is the Dizziness Handicap Inventory (DHI); secondary outcomes include the Dizziness and Anxiety Rating Scale (DARS), dizziness diaries and adverse event rate during the follow-up period. To test the *a priori* hypothesis that acupuncture improves dizziness by modulating posterior circulation hemodynamics and regional cerebral perfusion in vestibular-related brain regions, cerebral hemodynamics and brain imaging changes will be assessed using transcranial Doppler (TCD), structural magnetic resonance imaging (sMRI), and arterial spin labeling MRI (ASL-MRI) at baseline (Week 0) and post-treatment (Week 3). We specifically hypothesize that acupuncture will increase mean flow velocity (MFV) and reduce resistance index (RI) in the vertebral arteries, basilar artery, and posterior cerebral arteries, enhance regional cerebral perfusion in vestibular-related brain areas, and induce structural changes associated with vestibular compensation.

**Discussion:**

This study will provide robust evidence on the safety and efficacy of acupuncture for vertigo with PCIS. In addition, it will test a prespecified biological hypothesis that acupuncture may relieve dizziness by improving vertebrobasilar hemodynamics and regional perfusion within vestibular-related brain networks, with corresponding imaging changes associated with clinical improvement. This research aims to offer novel insights into acupuncture as a potential therapeutic approach for vertigo following PCIS.

**Clinical trial registration:**

[chictr.org.cn], identifier [ChiCTR2400087030].

## Introduction

Posterior circulation ischemic stroke (PCIS) with vertigo is one of the common types of central vertigo, accounting for approximately 20–30% of all ischemic strokes, and is frequently associated with severe dizziness and unfavorable functional recovery (1). While motor, speech, and cognitive recovery in stroke patients has received substantial therapeutic attention, chronic vertigo symptoms are frequently overlooked (2, 3). As an independent risk factor for stroke patients (4), vertigo reduces rehabilitation participation, disrupts rehabilitation progress, and even increases stroke recurrence and mortality rates, forming a vicious cycle that impairs overall post-stroke recovery (5).

Currently, for patients with PCIS accompanied by vertigo, clinicians typically use medications such as betahistine. Given the absence of disease-specific, evidence-based pharmacotherapy for vertigo associated with PCIS, we selected betahistine as the comparator in this trial. Although betahistine is most widely studied in peripheral vestibular disorders such as Ménière’s disease, it remains the most commonly prescribed agent for central vascular vertigo in clinical practice, particularly for patients with vertebrobasilar insufficiency. Betahistine acts as a partial agonist at H2 receptors and an agonist at H1 receptors, exerting significant vasodilatory effects on cerebral, coronary, and peripheral vessels, particularly the vertebrobasilar system, thereby increasing blood flow to the brain, heart, and peripheral tissues (6). Meanwhile, it acts as an H3 receptor antagonist to improve central vestibular compensation (7, 8). These dual mechanisms provide a biologically plausible rationale for its use in PCIS with vertigo. As no definitive first-line medication exists for this indication, betahistine represents the most pragmatic and clinically relevant choice to enable a direct comparison with acupuncture. However, achieving long-term control and improvement of vertigo symptoms with betahistine typically requires both high doses and prolonged administration (9). Clinical studies indicate that existing pharmacological treatments provide limited efficacy evidence for managing vertigo associated with PCIS, and symptoms frequently recur upon discontinuation of the medication (10). Additionally, polypharmacy in stroke patients may reduce adherence, increase drug resistance, and impose extra physical and psychological burdens, highlighting the critical need for safe, effective, and well-tolerated non-pharmacological interventions.

Acupuncture, recommended by the World Health Organization (WHO) as an alternative and complementary strategy for stroke management and rehabilitation (11), has shown promising efficacy and safety for post-stroke vertigo (12). However, most studies have primarily utilized acupuncture as an adjunct to western medical treatments (e.g., betahistine, flunarizine), which makes it difficult to clearly delineate the specific therapeutic effects of acupuncture itself. The clinical effectiveness of acupuncture has been questioned due to potential psychological or placebo effects, leading to concerns about the rigor of control designs in many studies. Overall, the clinical evidence supporting acupuncture for PCIS with vertigo remains limited and generally of low quality (13). Therefore, the present study is designed to provide novel insights in two key aspects. First, by independently establishing a sham acupuncture control group and a western medication control group, this trial aims to isolate the specific therapeutic effects of acupuncture as a standalone intervention, disentangling its intrinsic efficacy from non-specific factors such as concomitant drug effects or placebo responses. Second, a treatment expectation assessment will be incorporated to quantitatively evaluate the potential influence of psychological factors on treatment outcomes, an aspect seldom addressed in previous studies.

Pathophysiologically, normal brain function relies on stable metabolic processes, and stroke-induced cerebral metabolic imbalances and secondary structural changes (14). In clinical practice, post-stroke vertigo patients often experience dizziness that is not related to positional changes but rather to prolonged physical activity or sustained attention, associated with cerebral blood flow dysregulation and subsequent oxygen and glucose imbalance (15). Moreover, following ischemic strokes, structural changes in the brain, such as alterations in white matter integrity and gray matter volume, have been well-documented. Studies indicate that structural abnormalities, including infarct location and total lesion volume, may be closely linked to the occurrence of vertigo after stroke (16). In particular, lesions involving the vestibular nuclei, brainstem, cerebellum, or vestibulo-cerebellar structures may be especially relevant to dizziness symptoms (17). Nevertheless, in patients with PCIS, the severity and persistence of dizziness may not be fully explained by lesion topography alone. Residual vertebrobasilar hypoperfusion, altered neurovascular regulation, and secondary reorganization of vestibular-related brain regions may also contribute to symptom burden during recovery (18). Therefore, in the present trial, lesion location will be documented at baseline, while TCD, sMRI, and ASL-MRI will be used as complementary tools to characterize dynamic cerebral hemodynamics, regional perfusion, and structural changes associated with symptom persistence and recovery.

Hence, this study designed as a multicenter, large-sample randomized controlled trial aimed at: (1) Primary objective: To evaluate the specific efficacy and safety of manual acupuncture for PCIS with vertigo or dizziness by comparison with sham acupuncture. (2) Secondary objective: To compare the effectiveness and safety of manual acupuncture with western medication (betahistine) for PCIS with vertigo or dizziness. (3) Mechanistic objective: To test the *a priori* hypothesis that acupuncture alleviates vertigo by improving posterior circulation hemodynamics and regional cerebral perfusion in vestibular-related brain regions. Changes in TCD parameters, ASL-MRI perfusion, and sMRI structural markers will be evaluated and correlated with improvements in DHI scores.

## Patients and methods

### Study design

This randomized, three-armed, parallel-controlled, multicenter clinical trial was approved by the Medical Ethics Committee of the Affiliated Hospital of Chengdu University of Traditional Chinese Medicine (approval number: 2024KL-021) and registered with the China Clinical Trial Registration Center (registration number: ChiCTR2400087030; https://www.chictr.org.cn/), prior to the enrollment of the first participant. The protocol adheres to the SPIRIT statement. The future trail will carry out in accordance to the Declaration of Helsinki and follow the Consolidated Standards of Reporting Trials (CONSORT) guidelines (19), including the STRICTA extension for reporting interventions in clinical trials of acupuncture (20).

**Figure 1:**
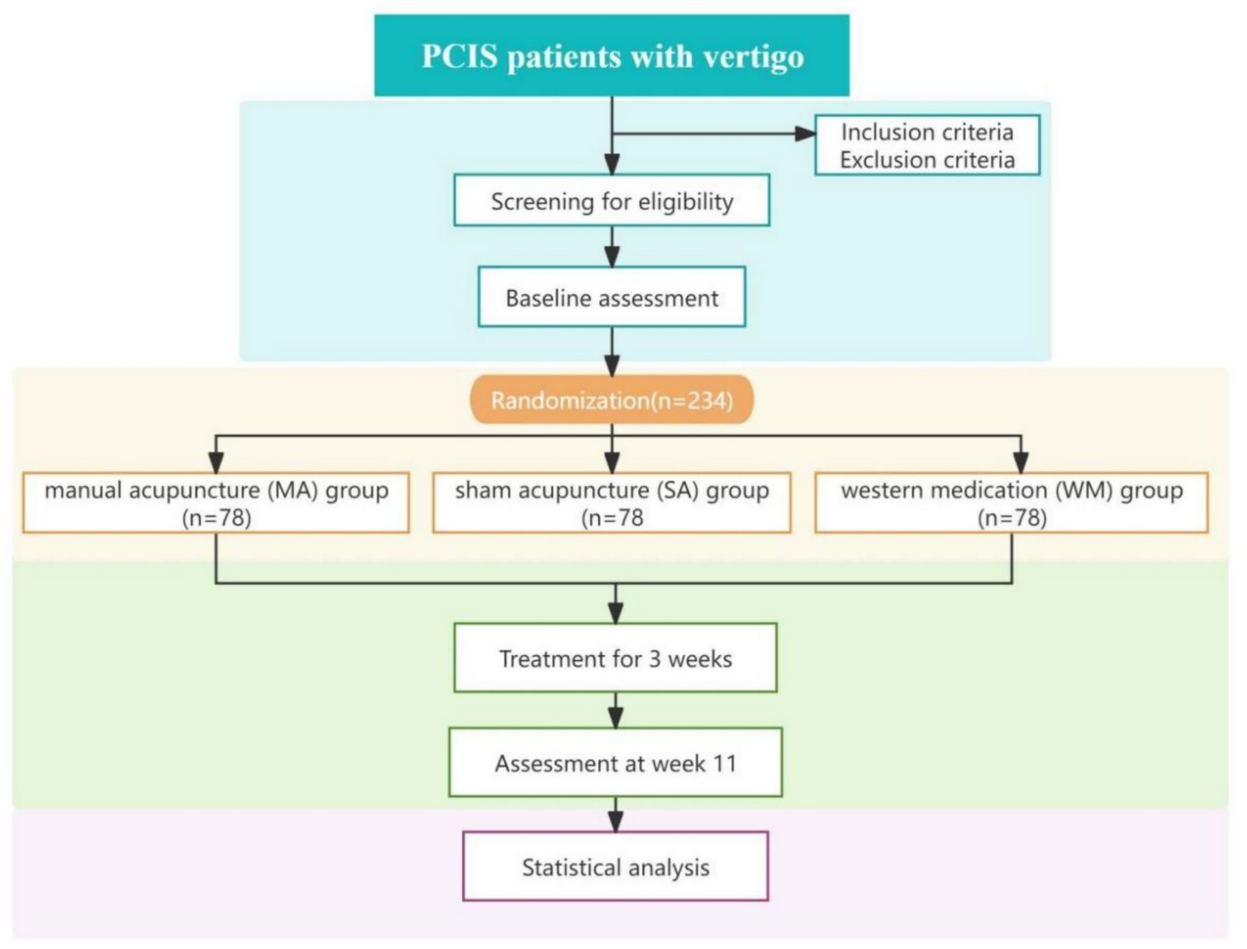
Research flow chart.

All participants are coming from 3 tertiary hospitals in Sichuan Province, China: the Affiliated Hospital of Chengdu University of Traditional Chinese Medicine, Chengdu Shuangliu District Hospital of Traditional Chinese Medicine and Chengdu Longquanyi District Hospital of Traditional Chinese Medicine. The study period comprised a 1-week baseline phase, a 3-weeks treatment phase, and an 8-weeks follow-up phase. A flow chart outlining the trial design is presented in Figure 1, while details on patient enrollment, intervention, and assessment times are shown in Table 1.

**Table 1:** Study schedule for data measurements.

Study period	Baseline	Allocation	Intervention period	Follow-up period
Time point (week)	−1	0	3	11
Enrolment and Baseline data:	Eligibility screen; informed consent; demographic characteristics; disease/combination medication history	Randomization		
Primary outcome:		DHI	DHI	DHI
Secondary outcomes:		DARS; dizziness diaries	DARS; dizziness diaries; adherence assessment	DARS; dizziness diaries
Objective hemodynamic/ imaging indicators:		TCDsMRIASL-MRI	TCDsMRIASL-MRI	
Other assessments:		Treatment expectations assessment	Safety assessment	Safety assessment

DHI, Dizziness Handicap Inventory; DARS, Dizziness and Anxiety Rating Scale; TCD, transcranial Doppler; sMRI, structural magnetic resonance imaging; ASL-MRI, arterial spin labeling MRI.

### Randomization and blinding

A random number will be generated with the R software (V.4.3.3) and concealed in opaque random envelopes. The envelopes will be prepared by a third party in advance, with the sequence number labeled on the surface and the random number and group allocation information sealed in. The random envelopes will be preserved by special personnel who are not involved in treatment and evaluation. Participants are randomly assigned in a ratio of 1:1:1 to manual acupuncture (MA) group, sham acupuncture (SA) group, and western medication (WM) group.

Given the use of two distinct acupuncture techniques in this trial, the acupuncturist will be aware of the group allocation for each participant. However, identical auxiliary devices will be used in both the acupuncture and sham acupuncture groups to maintain blinding for participants. Participants in both groups will remain unaware of their specific group assignment. Furthermore, outcome assessors and statisticians will be blinded to group allocations throughout the study to minimize bias. To maintain blinding, all researchers will undergo identical training prior to the trial, and treatments will be administered in separate rooms for each participant. It should be noted that participants in the medication group will not be blinded.

### Participants

#### Inclusion criteria

Participants with dizziness or vertigo meeting the WHO criteria (21) and international Bárány association standards (18) for PCIS will be included. Symptoms may include ataxia, unilateral or bilateral visual, motor or sensory disturbances, double vision, dysarthria or swallowing impairment. Patients must present with TCD or magnetic resonance imaging (MRI) results indicative of vertebrobasilar insufficiency.

Additionally, participants must meet all of the following criteria: 1) in the non-acute stage (defined as >14 days after stroke onset per international stroke guidelines) with stable condition and no acute exacerbation; 2) aged 30 to 80 years, both sexes; 3) dizziness or instability symptoms lasting ≥ 1 week and occurring ≥ 2 times; 4) vertigo attributable solely to central vestibular dysfunction caused by posterior circulation infarction, with no otogenic, ophthalmogenic, or other systemic causes; 5) Basel Scale Index rating scale score of more than 40; 6) able to complete or assist in completing a dizziness diary; 6) willing to cooperate with the study and sign an informed consent form.

#### Exclusion criteria

Patients who meet any of the following conditions will be excluded: 1) dizziness confirmed to be caused by other diseases or trauma; 2) history of central nervous system disorders (e.g., cerebral hemorrhage, intracranial infection) within the past 6 months; 3) severe underlying primary diseases affecting the cardiovascular, respiratory, hepatic, renal, hematopoietic systems, or serious dysphrenia; 4) severe skin infections at acupuncture point; 5) pregnant or lactating women; 6) currently participating in another clinical trial or having participated in one within 3 months prior to recruitment.

### Patient recruitment

Recruitment strategies include poster, online platforms, and rehabilitation and neurology departments inpatients. Potential patients will be informed about the study specifics, including the purpose, subgroup allocation, interventions, treatment period, benefits, and potential risks. A neurology specialist will assess and diagnose interested patients to determine their eligibility. Eligible individuals who voluntarily choose to participate will be required to sign a written informed consent form prior to the start of the study. All patient information will be kept confidential. Participants have the right to withdraw from the study at any stage, with reasons for withdrawal being thoroughly documented.

### Intervention and comparison

All patients will receive guideline-based interventions (22, 23) for the secondary prevention of ischemic stroke which include: 1) management of risk factors such as blood pressure and blood glucose; 2) targeted treatments: administration of aspirin for antiplatelet therapy or warfarin for anticoagulation, as appropriate, along with atorvastatin for lipid regulation and plaque stabilization; 3) patient education on dietary and lifestyle modifications. Specific interventions for each group are as follows: the MA group will receive manual acupuncture in addition to standard secondary prevention treatment; the SA group will receive sham acupuncture alongside standard secondary prevention treatment; and the WM group will receive betahistine (24) in conjunction with standard secondary prevention treatment.

### Manual acupuncture (MA) group

**Figure 2:**
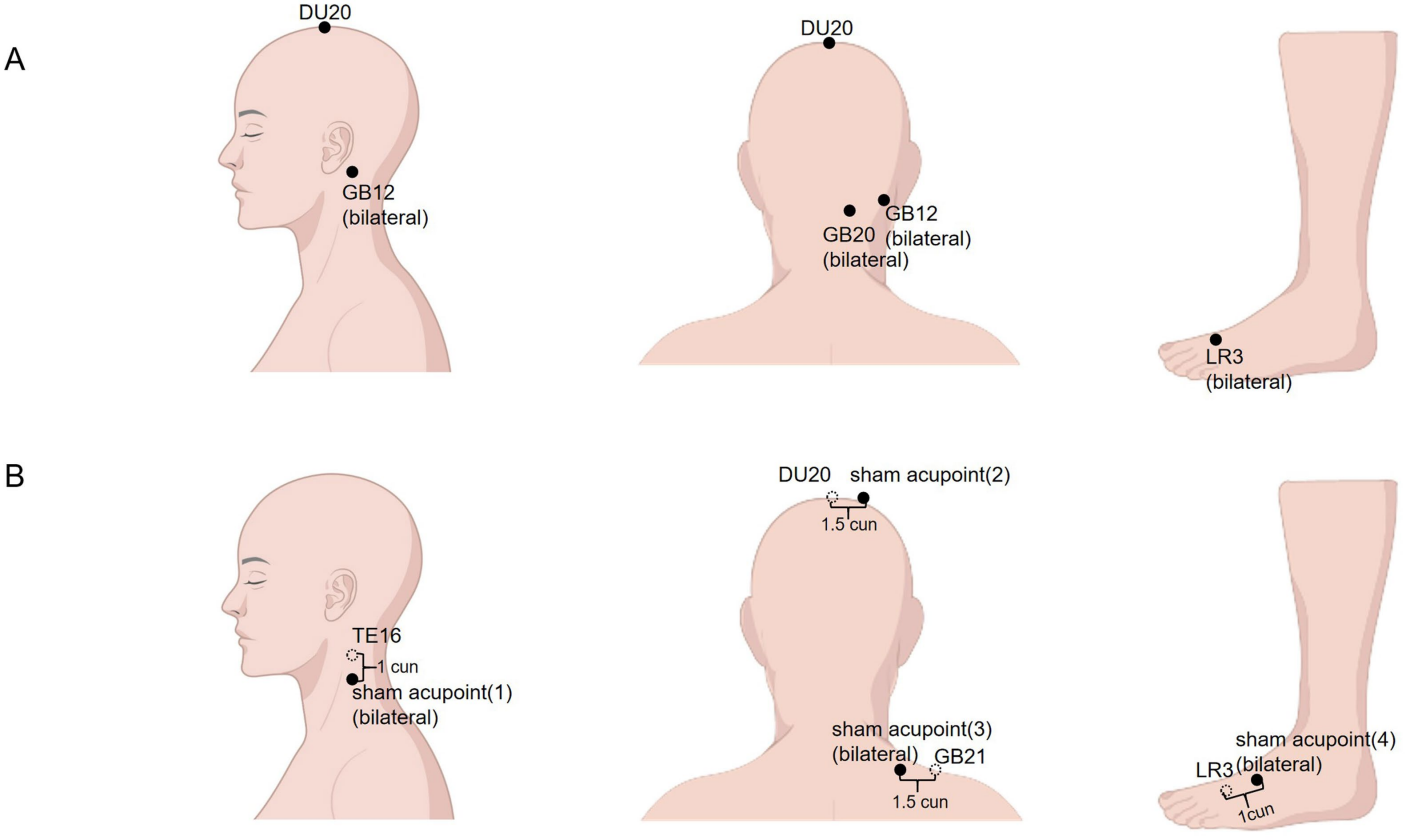
Location of real acupoints and sham acupoints **(A,B)**. DU, Du Mai; GB, Gallbladder Meridian; LR, Liver Meridian; TE, Triple Energizer Meridian.

The selected acupuncture points—Baihui (DU20), Fengchi (GB20), Wangu (GB12), and Taichong (LR3)—were determined based on preliminary literature review and consensus among acupuncture experts for this study. Detailed descriptions of each acupoint are provided in Table 2 and illustrated in Figure 2. All acupuncturists are licensed and have a minimum of 3 years of clinical experience. Prior to the trial, all acupuncturists will undergo training on acupoint positioning and manipulation techniques. Each sub-center will be assigned one acupuncturist who will be responsible for treating all patients at that center.

**Table 2:** Locations of acupoints.

Acupoints	Locations
Baihui (DU20)	On the median line of the head, 5 inches superior to the anterior hairline, at approximately the middle of the connecting line between the two auricular tips.
Fengchi (GB20)	On the posterior neck, inferior to the occipital bone, in the depression between the origins of sternocleidomastoid and the trapezius muscles.
Wangu (GB12)	On the posterior aspect of the head, in the depression between the mastoid process and the inferior nuchal line of the occipital bone.
Taichong (LR3)	0.5 to 0.8 finger breadths laterally to the dorsum of the foot, in the distal depression of the angle proximal between the 1st and 2nd metatarsa.

**Figure 3:**
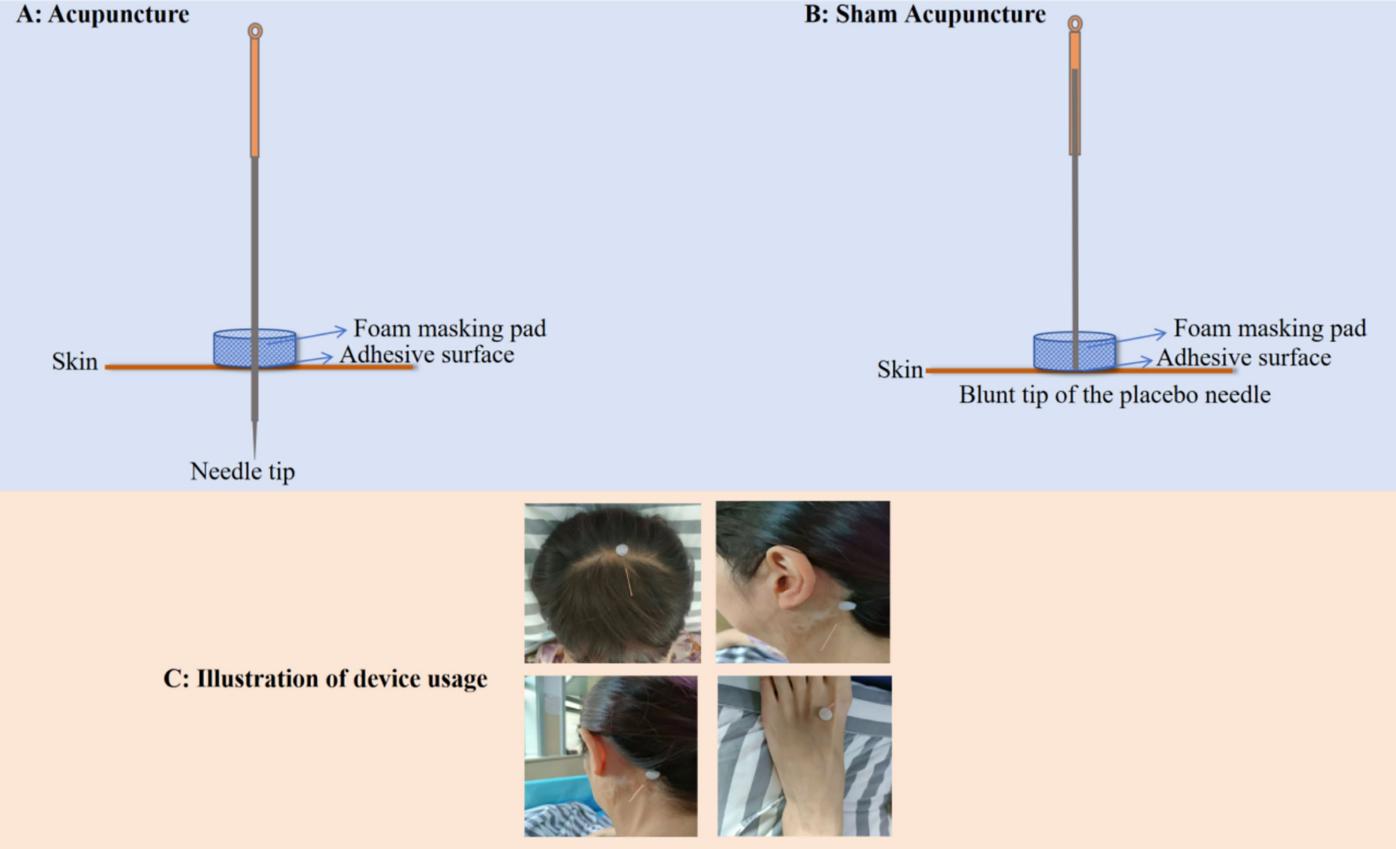
Illustration of acupuncture. **(A)** Real acupuncture device; **(B)** Sham acupuncture device; **(C)** Usage of device.

During the acupuncture process, patients will maintain a semi-recumbent position. The acupuncturist will sterilize the skin around the acupoint using 75% alcohol, then stick a foam base to the acupoint and insert the needle through the foam base (Figure 3A). For DU20, a 1 cun needle (0.30 × 25 mm, Suzhou Hua Tuo, China) will be inserted laterally to a depth of approximately 0.5–0.8 cun at a 15° angle relative to the skin. For GB20, GB12, and LR3, needles of the 1 cun (0.30 × 25 mm, Suzhou Hua Tuo, China) will be inserted to a depth of approximately 0.5–1.0 cun. Needles will be retained for 30 min, with manipulation (lifting, thrusting, twisting, and rotating) performed every 10 min to achieve the sensation of “De Qi.” Upon needle removal, a dry-sterilized cotton ball will be used to gently press the needled area to prevent bleeding. Each patient will undergo a 3-week acupuncture treatment regimen, receiving five sessions per week for a total of 15 sessions.

### Sham acupuncture (SA) group

The sham acupuncture procedure closely mirrors that of manual acupuncture. The procedure will be also performed using Streitberger placebo needles which will be applied at non-acupoint locations, approximately 1–1.5 cun lateral to the actual acupoints along the meridians (Figure 2 and Figure 3B). No lifting, thrusting, or rotating manipulations will be performed, and no “De Qi” sensation will be elicited. The patients may experience a sensation of pain but not skin puncture. To address ethical considerations, participants in the sham acupuncture group will receive a free compensatory acupuncture treatment at the conclusion of the trial. After randomization, participants receive treatment five sessions per week for 15 sessions for three consecutive weeks.

### Western medication (WM) group

Betahistine mesilate tablets (6 mg per tablet; Eisai (China) Pharmaceutical Co., Ltd.; National Medical Products Administration No. H20040130) are used, one tablet each time, three tablets per day, after meals. The treatment was administered continuously for 5 days per week over a 3-week period.

### Outcome measurements

The outcome measures and evaluation time points are summarized in Table 1.

#### Primary outcome

The Dizziness Handicap Inventory (DHI) is the most widely used self-reported measure for assessing dizziness across three domains (25): physical (DHI-P, maximum score of 28), emotional (DHI-E, maximum score of 36), and functional (DHI-F, maximum score of 36). Each item is scored as 0 (no), 2 (sometimes), or 4 (yes). The total score, ranging from 0 to 100, is calculated by summing the scores of all items, with higher scores indicating more severe the dizziness disorder. These outcome measures will be repeatedly assessed at weeks 0, 3, and 11 by a researcher who is blinded to participant allocation.

#### Secondary outcomes

Secondary outcomes included the following eight items: 1) change in the Dizziness and Anxiety Rating Scale (DARS) at 0, 3, and 8 weeks; 2) changes in the total frequency of vertigo episodes over weeks 1, 3, and 8; 3) changes in the average duration of vertigo episodes over weeks 1, 3, and 8; 4) changes in the average severity of vertigo episodes over weeks 1, 3, and 8; 5) changes in the average impact on quality of life scores of vertigo episodes over weeks 1, 3, and 11.

### DARS

The DARS evaluates the degree of balance disorder and disorientation caused by dizziness. The DARS consists of 6 items, each rated on a 7-point scale: asymptomatic (0), very mild (1), mild (2), mild to moderate (3), moderate (4), moderate to severe (5), and severe (6). The total possible score ranges from 0 to 36, with higher scores indicating more severe dizziness.

### Dizziness diaries

Participants will be required to maintain a dizziness diary, recording each episode of dizziness in detail. For each episode, participants will document the attack duration, date of occurrence, vertigo intensity, impact on daily life, and any acute medications taken. Vertigo intensity will be evaluated using a VAS (0 to 10). This detailed record will facilitate the comprehensive assessment of dizziness frequency, severity, and its impact on daily activities throughout the study period.

### TCD, sMRI and ASL-MRI outcome measures

This trial will concentrate on the following TCD, sMRI and ASL-MRI indicators (week 0, and week 3). For TCD, assessment includes measuring the mean flow velocity (MFV) and resistance index (RI) in the bilateral middle cerebral arteries (MCA), anterior cerebral arteries (ACA), posterior cerebral arteries (PCA), vertebral arteries (VA), and basilar artery (BA) for TCD. For sMRI, evaluations will encompass voxel-based whole-brain voxel-mirrored homotopic connectivity (VMHC), cortical thickness, gray matter volume (GMV), white matter volume (WMV), and total brain volume (TBV). These comprehensive assessments aim to provide detailed insights into cerebrovascular function and brain structural changes over the study period. For ASL-MRI, the following image data will be acquired: high-resolution 3D T1-weighted images for delineation of gray and white matter regions of interest (including deep gray matter structures), to support ASL data analysis and spatial normalization to a standard brain atlas. The total scanning time per MRI session will be within 15 min.

### Treatment expectations assessment

Before the treatment, participants in each group will be asked, “How do you perceive the final efficacy of the treatment?” Participants will select from one of five options: 1) highly effective (symptom improvement >75%); 2) moderately effective (symptom improvement 50%–75%); 3) slightly effective (symptom improvement 25%–50%); 4) no change (symptom improvement 0–25%). Responses indicating “highly effective” will be categorized as high expectations, while all other responses will be categorized as low expectations.

To minimize expectation bias, outcome assessors and statisticians will remain fully blinded to group allocation throughout the study. In addition, treatment expectation level will be incorporated as a covariate in the statistical analysis of the primary outcome (DHI) to adjust for its potential confounding influence on subjective symptom reports. To further ensure objectivity, all assessments will be performed using standardized procedures, and expectation evaluation will be conducted by a researcher blinded to treatment assignment.

### Safety assessments

Throughout the study, participants may encounter adverse events (AEs) related to acupuncture, including needle sickness, subcutaneous hemorrhage, hematoma, severe pain, infection, needle breakage, rash, and other symptoms. Additionally, during the administration of betahistine mesylate tablets, patients may experience gastrointestinal symptoms such as nausea, vomiting, and diarrhea, as well as allergic reactions like rash. All AEs will be meticulously documented in the Case Report Form (CRF), detailing the occurrence time (transient or persistent), severity (mild, moderate, or serious), possible causes (needle-related, medication-related, or non-needle-related), symptoms, duration, treatment measures, and resolution time. Prompt and appropriate treatment will be provided to participants experiencing mild to moderate AEs, with continuous monitoring by the attending acupuncturist. For serious AEs, immediate medical advice will be given within 48 h, and these events will be reported to the principal investigator and the research ethics committee within 24 h. Clinicians will determine whether to discontinue the trial based on the participant’s condition.

To evaluate differences in AEs between groups, statistical analyses using the Chi-square test or Fisher’s exact test will be conducted. This analysis aims to identify significant variations in the occurrence of adverse reactions between the treatment and control groups, thereby providing valuable insights into the safety of the interventions.

### Sample size calculation

As reported in previous studies (8), the mean DHI score for patients treated with betahistine mesilate was 30. For the purpose of sample size estimation, we adopted the clinically meaningful difference derived from our primary comparison: manual acupuncture versus sham acupuncture, assuming a mean DHI score of 21 in the manual acupuncture group, 33 in the sham acupuncture group, with a standard deviation of 20. A 12-point difference in DHI total score between manual acupuncture and sham acupuncture was regarded as clinically meaningful, since the minimal clinically important difference (MCID) for DHI total score has been established as 11 points (26). The trial will include three groups in a 1:1:1 ratio. Using a twotailed test with a category 1 error of 0.05 and a statistical efficacy of 90%, One-Way Analysis of Variance (ANOVA) F-Tests was employed by PASS software (Power Analysis and Sample Size, version 15). The calculations indicate that a minimum of 66 participants per group is required, totaling 198 participants across all groups. Accounting for an estimated drop out rate of 15%, the total sample size should be increased to 234 participants, with at least 78 participants per group.

### Data management and confidentiality

The case report form (CRF) for each participant will be promptly completed and subsequently reviewed and entered into a secure database by an individual not involved in the treatment. To ensure confidentiality, all personal information, including names, phone numbers, and addresses, will be anonymized to prevent any disclosure. The CRF will include key data points such as observation time points, imaging time points, outcome measures, adverse events, and safety assessments. Researchers are required to complete the CRFs accurately and in a timely manner according to the specified protocol. All participant data will be securely stored in a locked cabinet by the research team and retained for at least 5 years after publication in a medical journal. This ensures compliance with data protection regulations and allows for future reference and verification.

### Statistical analysis

Analyses for this study will be conducted in accordance with both the intention-to-treat (ITT) principle and the per-protocol set (PPS) approach. The ITT analysis will include all randomized participants, and the missing data will be imputed using the Last Observation Carried Forward (LOCF) method, with the most recent observed data carried forward for filling. The PPS will include participants who have completed at least 80% of the prescribed treatment. SPSS 23.0 software will be used for statistical analysis. Quantitative indicators are described using mean, standard deviation, median, 25th percentile and 75th percentile. Categorical indicators are described using frequencies and percentages.

To control the Type I error caused by multiple comparisons, the Bonferroni correction will be adopted for pairwise comparisons of the primary outcome (DHI total score) among the three groups (manual acupuncture vs. sham acupuncture, manual acupuncture vs. western medication, sham acupuncture vs. western medication), with the adjusted significance level set at *p* < 0.017 (0.05/3); for secondary outcomes (DARS scores, dizziness diary indicators including attack frequency, duration and severity), the false discovery rate (FDR) method will be used for multiple comparison correction to balance the Type I and Type II error rates in exploratory analysis of multiple secondary indicators.

Since this study is a multicenter clinical trial, sub-center and corresponding baseline values were incorporated as covariates in the generalized linear model for analysis. Additionally, binary logistic regression was employed to analyze the impact of high versus low expectations on the primary efficacy endpoint across groups. The dependent variable in this analysis was response status, defined as participants showing at least a 50% reduction in total DHI scale score from baseline after 3 weeks of treatment (referred to as responders). The independent variable was the level of expectation (high or low).

Neuroimaging data (sMRI and ASL-MRI) will be analyzed with professional software and standardized methods, and all analyses will be completed by an experienced radiologist who is blinded to the participant group allocation. sMRI data will be preprocessed and analyzed using SPM12 (Statistical Parametric Mapping 12) and FSL (FMRIB Software Library); the preprocessing steps include skull stripping, spatial normalization, tissue segmentation (gray matter, white matter, cerebrospinal fluid) and 8 mm Gaussian kernel smoothing, and then voxel-based morphometry (VBM) and voxel-mirrored homotopic connectivity (VMHC) will be used to analyze group differences in gray matter volume (GMV), cortical thickness and interhemispheric functional connectivity. ASL-MRI data will undergo motion correction, distortion correction and arterial transit time (ATT) correction to calculate cerebral blood flow (CBF) maps; region of interest (ROI) analysis will be performed on vestibular-related brain regions (vestibular nuclei, brainstem, cerebellum, posterior cerebral cortex) to compare group differences in regional CBF, and whole-brain voxel-wise analysis will be conducted to explore potential brain regions with altered perfusion associated with vertigo improvement.

### Quality control

The trial protocol has been reviewed and revised by experts in acupuncture, neurology, and statistics. Standard operating procedures (SOPs) will be developed in advance, and training sessions will be conducted prior to the study commencement to provide standardized training for researchers at each center. This ensures that all researchers are familiar with the study process and specific implementation details. Trial participants should remain consistent, and both recording methods and evaluation criteria must be standardized to ensure uniformity in scale assessments, data management, and CRF completion. Data monitors will conduct quality control checks every 3 months at each research center to ensure strict adherence to all elements of the study protocol and the accuracy of complete information. Quality control analysis reports will be generated following each inspection.

## Discussion

Recurrent vertigo episodes in PCIS patients increase recurrence and mortality rates, creating a cycle of worsening health. This not only heightens the health burden on individuals but also imposes significant economic pressures on healthcare systems and society.

The DHI is widely acknowledged as the gold standard for assessing patients with vertigo symptoms, with well-documented validity, reliability, and reproducibility in clinical trial settings. This comprehensive questionnaire evaluates the precipitating physical factors associated with vertigo and assesses the functional and emotional impacts of vestibular impairment. The primary aim of the DHI is to quantify the disability and impairment caused by vertigo, providing insights into its effects on patients’ daily activities (27). The DHI consists of three main components: physical symptoms of dizziness and vertigo, vestibular dysfunction, and emotional impact, and serves as a valuable tool in both clinical practice and research for evaluating the multidimensional impact of vertigo on patients’ quality of life. Accordingly, the DHI was selected as the primary outcome in this trial because it represents the most patient-centered and clinically relevant measure for capturing the overall burden of dizziness in patients with posterior circulation ischemic stroke. This choice is consistent with international consensus and core outcome recommendations for clinical trials involving vertigo.

Pearson’s analysis showed that the DHI and DARS had a good correlation and were consistent in the assessment of vertigo patients, and all of them could be used to assess the degree of vestibular function impairment (28). The DARS scale is comprehensive, highly reliable, easy to understand. In addition, dizziness diaries are helpful for analyzing patients’ subjective symptoms: they enable us to confirm whether the characteristics of the attacks meet the diagnostic criteria, and they also facilitate more effective monitoring of treatment outcomes, which is especially important due to the non-specific episodic nature of the disorder. Furthermore, the use of dizziness diaries has enabled a prospective observational approach in this study (29). Nevertheless, subjective patient-reported outcomes are inevitably affected by individual symptom perception, emotional state, and psychological factors. In acupuncture trials, these subjective responses are particularly prone to the influence of treatment expectation and non-specific contextual effects, which may partially contribute to perceived improvement. Although we quantitatively assessed treatment expectation to explore its potential influence, such assessment alone cannot completely exclude confounding from expectancy or placebo-like effects. Thus, caution should be exercised when interpreting the observed treatment effects, with due consideration given to the inherent limitations of subjective outcome measures.

To reduce reliance on subjective patient-reported outcomes, this trial places strong emphasis on objective hemodynamic and imaging biomarkers, including TCD, sMRI, and ASL-MRI. However, research on the effects of acupuncture on cerebral blood flow and brain imaging remains relatively limited and requires further development and refinement (30). At present, the underlying cerebral mechanisms associated with acupuncture in the treatment of vertigo following posterior circulation ischemic stroke have not been fully elucidated, highlighting the need to advance related research. Therefore, this study not only evaluates the therapeutic efficacy of acupuncture for PCIS with vertigo, but also employs TCD, sMRI and ASL-MRI to provide a more in-depth assessment of the potential hemodynamic mechanisms and subtle structural changes associated with post-stroke vertigo. By doing so, this approach not only provides a scientific basis for acupuncture in treating PCIS with vertigo, but also explores its underlying therapeutic mechanisms, offering new insights into clinical management and potentially improving treatment outcomes.

Both pragmatic and explanatory randomized controlled trials play important roles in evaluating healthcare interventions. Explanatory trials are designed to establish the absolute efficacy of a treatment intervention (31). In acupuncture research, the primary goal of an explanatory trial is to determine whether acupuncture is superior to a placebo. A sham acupuncture control in clinical trials should meet three key criteria: (1) the sham intervention has little or no specific therapeutic effect; (2) the needles are applied at non-effective locations; and (3) participants are unable to distinguish between the sham and real acupuncture treatments. To rigorously satisfy these criteria and resolve the inconsistent efficacy outcomes reported in previous sham acupuncture-controlled trials, our sham acupuncture protocol was developed based on evidence-based principles for device selection and non-acupoint localization specifically tailored to acupuncture RCTs for brain function-related disorders (32). For such disorders (including PCIS with vertigo), the Streitberger non-penetrating placebo needle is the preferred sham device. Non-acupoint sites are also recommended to be located in non-meridian regions adjacent to genuine acupoints; this approach can strengthen the placebo effect in the control group and lower the risk of false-negative outcomes in clinical trials (33). Additionally, the specific localization of our non-acupoint sites (1–1.5 cun lateral to the actual acupoints along the meridians) was determined in accordance with standardized non-acupoint selection criteria validated in relevant reference literature (34). Considering these principles, we selected a non-meridian, non-acupoint location adjacent to the acupoints used in the acupuncture group for the sham intervention. To enhance blinding, we will use a blunt-tipped placebo needle that mimics the sensation of real acupuncture without penetrating the skin. Upon contact with the skin, the tip of the placebo needle creates a slight pricking sensation, but does not pierce the skin. Combined with the perceived insertion and retention, participants are likely to perceive this as genuine acupuncture, thereby improving the effectiveness of blinding. Although non-acupoints adjacent to meridians were adopted for sham administration, potential mild physiological effects from such locations cannot be fully excluded. This sham design represents the most feasible and widely validated approach for brain-related disorders, but residual non-specific effects may still exist and should be acknowledged when interpreting specific efficacy.

Given that betahistine is commonly used in the clinical management of vertigo following PCIS, we have also included a medication control group treated with this drug, allowing for a comparative evaluation of the efficacy of acupuncture versus standard pharmacological therapy. While betahistine is predominantly indicated for peripheral vestibular disorders, its inclusion as an active comparator in this trial is methodologically sound and clinically informative for central vascular vertigo. This choice does not imply an assumption of superior efficacy but rather reflects real-world clinical practice, where no guideline-directed pharmacotherapy exists for PCIS with vertigo. Using the most widely adopted clinical agent as a comparator enables a pragmatic effectiveness comparison rather than an explanatory efficacy trial, which is more generalizable to routine care. Furthermore, our three-group design with true acupuncture, sham acupuncture, and betahistine permits simultaneous dissection of specific needling effects, non-specific placebo responses, and the actual clinical performance of standard care. Even if betahistine shows only mild or placebo-like effects in this study population, our trial will still produce high-quality evidence to clarify the added value of acupuncture and support evidence-based clinical decisions for these patients.

Notably, the present three-arm trial was established with a hierarchical study objective design to clearly differentiate explanatory from comparative effectiveness purposes. The primary comparison between manual acupuncture and sham acupuncture was specifically designated to evaluate the specific efficacy of acupuncture, whereas comparisons between acupuncture and western medication were defined as secondary analyses. This clearly predefined hierarchical structure minimizes potential ambiguity in the interpretation of study outcomes and reinforces the scientific validity and rigor of the trial design.

Necessarily, this study also has several limitations. First, participants in the western medication group could not be blinded due to the nature of oral intervention, whereas those in acupuncture groups were blinded. This incomplete blinding may introduce expectation bias and affect the objectivity of subjective outcomes. Future trials are expected to use a double-dummy design to achieve full blinding across all groups. Second, this study focused primarily on changes in cerebral blood flow and brain structure, without exploring potential cerebral functional mechanisms in PCIS patients with vertigo. Third, subjective outcome assessments rely on participant self-report which may be affected by individual perception and recall bias due to the lack of objective vestibular function tests. Fourth, the follow-up duration is relatively short and fails to capture the long-term therapeutic efficacy and recurrence risk of vertigo symptoms in PCIS patients.
